# Host Defense Peptide-Mimicking Polymers and Polymeric-Brush-Tethered Host Defense Peptides: Recent Developments, Limitations, and Potential Success

**DOI:** 10.3390/pharmaceutics13111820

**Published:** 2021-11-01

**Authors:** Hashem Etayash, Robert E. W. Hancock

**Affiliations:** Centre for Microbial Diseases and Immunity Research, Department of Microbiology and Immunology, University of British Columbia, 2259 Lower Mall Research Station, Vancouver, BC V6T 1Z4, Canada; hashem@hancocklab.com

**Keywords:** host defense peptides, antimicrobial polymers, polymer brush, biofilms, antibiotic resistance

## Abstract

Amphiphilic antimicrobial polymers have attracted considerable interest as structural mimics of host defense peptides (HDPs) that provide a broad spectrum of activity and do not induce bacterial-drug resistance. Likewise, surface engineered polymeric-brush-tethered HDP is considered a promising coating strategy that prevents infections and endows implantable materials and medical devices with antifouling and antibacterial properties. While each strategy takes a different approach, both aim to circumvent limitations of HDPs, enhance physicochemical properties, therapeutic performance, and enable solutions to unmet therapeutic needs. In this review, we discuss the recent advances in each approach, spotlight the fundamental principles, describe current developments with examples, discuss benefits and limitations, and highlight potential success. The review intends to summarize our knowledge in this research area and stimulate further work on antimicrobial polymers and functionalized polymeric biomaterials as strategies to fight infectious diseases.

## 1. Introduction

The rapid rise and spread of multidrug-resistant pathogens alongside the dwindling rate of antimicrobial drug development threaten global health and jeopardize economic stability [1,2,3]. Antibiotics are becoming increasingly ineffective, leading to an escalation in persistent illnesses and disabilities and an increasing rate of mortalities [4,5,6]. More than 700,000 deaths from antimicrobial resistance occur worldwide every year [7], while antibiotic failure in treating sepsis, for example, contributes to ~11 million deaths annually [8]. In the United States alone, drug resistance has resulted in >35,000 deaths [4], while failing antibiotics in sepsis treatment has led to >200,000 deaths. The ability of bacterial pathogens to develop biofilms has also exacerbated the antibiotic resistance crisis, causing notorious infections of tissues and implanted medical devices that are extraordinarily difficult to treat [9,10]. Biofilms are responsible for approximately 65–80% of all clinical disorders associated with infections, and unfortunately, there are no specific antibiofilm therapies to treat such infections [10]. Despite the substantial progress in antimicrobial drug research, drug resistance is still on the rise, and many diseases caused by resistant “superbugs” remain challenging to cure.

**Figure 1:**
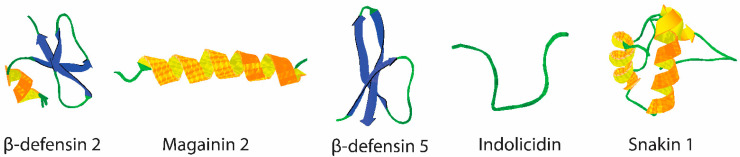
Examples of HDPs with diverse structures. The molecular models were reproduced from the RCSB Protein Databank (http://www.rcsb.org/pdb/home/home.do). β-defensin 2, 1W2E; magainin 2, 2MAG; α-defensin 5, 1ZMP; Indolicidin, 1G89; Snakin 1, 5E5Q.

HDPs were introduced in the early 1980s [11] as promising small molecules to replace failed/failing antibiotics in treating infectious diseases. All multicellular organisms naturally express these molecules as innate immune response elements that synchronise multiple tasks in and outside the cells [12,13]. Examples of the putative functions of HDPs include (1) eliminating bacterial growth through the direct antimicrobial activity without generating resistance [14,15,16], (2) inhibiting and eradicating biofilms by promoting dispersal and abolition through inhibition of biofilm-specific signalling pathways [17,18,19,20,21], and (3) modulating the innate immune responses through various mechanisms to indirectly resolve infections and inhibit potentially harmful inflammation [13,22,23,24]. As a result of these multifaceted functionalities, HDPs have recently re-emerged again as a new anti-infective group that captures a wide range of potential practical applications, including the treatment of mild and chronic infections [25] and inflammatory disorders [26], wound healing [27], tissue repair [28,29], the protection of implanted device [30], and many others [31]. Representative examples of the backbone structures of HDPs are shown in Figure 1. 

Structurally, HDPs are short-chain amphipathic molecules (usually between 10–50 amino acids) with cationic (charges, +2 to +9) and hydrophobic (proportion generally 30–65%) residues [31,32,33]. The conventional concept of the mode of action was that the cationic groups drive peptide adherence to the anionic surfaces of bacterial membranes, enabling insertion into the membrane driven by clusters of hydrophobic residues, which perturbs the integrity of the cell membrane. This was initially proposed to cause membrane damage and permeabilization, leakage of cellular components, and consequent cell death [31,32,34]; however, recent studies have suggested that such a mechanism is not by any means the only possible or even the major way in which many or most peptides kill cells. In contrast to prokaryotic cells, it was suggested that eukaryotic cells have a partially negatively charged cell membrane, thus decreasing binding of cationic HDPs and driving selectivity [35,36]; however, this too is not really correct since many of these peptides have the properties of cell-penetrating peptides and can freely translocate across eukaryotic membranes [37,38]. It is important to appreciate that, although membrane perturbation is often associated with the mechanism of action of HDPs, there is significant convincing evidence that HDPs have many other actions against bacteria. Indeed, it has been suggested that HDPs likely act in a multi-modal fashion to attack various targets [21,25,39,40]. For example, such peptides have been reported to target membrane-associated enzymes (e.g., inhibiting cell wall biosynthesis by sequestering the microbe-specific lipid receptor lipid II), inhibit cell division, inhibit macromolecular synthesis (protein, RNA or DNA synthesis by binding to intracellular targets, including nucleic acids), or target synthesis of other bacterial cell macromolecules, heat shock proteins, and others [21,39]. Indeed, the antibacterial activity of HDPs is far more complex than we thought, and further studies are needed in this area. In addition, since the interaction of HDPs with various targets is fairly non-specific (binding to complementary anionic and hydrophobic regions of the targets), it is unlikely we will see the development of bacterial resistance, as described for antibiotics. Since a complete discussion of the mechanism of action of HDPs is beyond the scope of this article, we refer the readers to the many articles that provide an in-depth discussion [23,31,41,42,43]. 

The benefits of HDPs over antibiotics and many other chemical compounds as a new generation of antimicrobial agents rely on their multifaceted functionality; broad spectrum of activity, including most existing antibiotic-resistant superbugs; and low development of bacterial resistance due to their multiple bacterial targets and the rapid bactericidal effects. Nevertheless, despite these benefits, the progression of HDPs into viable drug candidates is yet to be achieved. Their progress to clinical use has been languid and hampered by several constraints, including their inhibition by physiological concentrations of salts and anionic polymers, such as glycosaminoglycans; their susceptibility to proteases and peptidases that abound at infection sites; and (largely unknown) toxicity at higher concentrations as well as their cost and issues with large-scale production [44,45]. For this reason, several mimics of HDPs have been proposed and developed to address these shortcomings, such as all-D amino acid peptides [46,47], β-peptides [48], peptoids [48,49], peptide-mimicking polymers [50], and others [51], which were to some extent successful in reproducing biological properties similar to those of HDPs. This review explicitly discusses HDP-inspired antimicrobial polymers and addresses their design principles, recent developments, limitations, and future development. It also highlights the concept of engineering polymer brushes with HDPs in medical devices and implants to defeat infections and biofilms. 

## 2. HDP-Mimicking Polymers

Inspired by the unique properties of HDPs, cationic antimicrobial polymers (exchangeably stated as HDP-mimicking polymers) were conceived to overcome certain inherited limitations of HDPs and generate clinically accepted antimicrobials. These HDP-mimicking polymers are designed to retain the critical structural features of HDPs, thus generating similar or more biologically active compounds while being less expensive and easier to manipulate and produce on large scales. 

### 2.1. Fundamental Structural Design Principle

**Figure 2:**
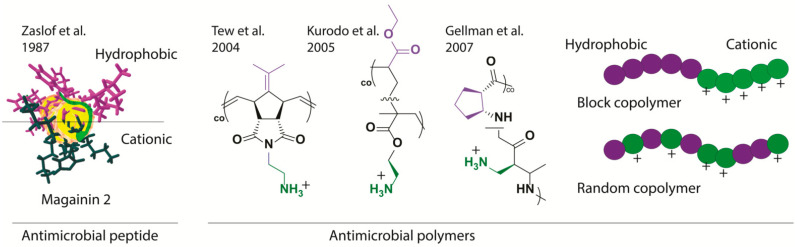
Evolution of antimicrobial polymers from HDPs. Magainin 2, as an example of an HDP, shows the hydrophobic and cationic domains. In the centre are examples of antimicrobial polymers, and on the right are examples of the described strategies of monomers connectivity in polymers, (block and random), where green and purple circles represent cationic and hydrophobic monomers, respectively.

The theoretical structural design of HDP-mimicking polymers is based on combining the properties of HDPs with the structural benefits of polymer disinfectants to create mimics with amphiphilicity and antimicrobial functions against a broad range of microorganisms [52]. As described earlier, HDPs are generally rich in cationic amino acids (e.g., arginine and lysis) and amino acids with hydrophobic side chains (e.g., tryptophan, phenylalanine, tyrosine, leucine, isoleucine, and valine), which give them an amphiphilic nature that is crucial to their action and promotes their attachment to the bacterial surface/membranes (Figure 2, magainin 2). By analogy, the cationic polymers consist of two main functional components/monomers: the first contains cationic functional groups, and the second has hydrophobic functional groups (see examples in Figure 2 from references [53,54,55]). These two monomers are connected in various ways (e.g., random or block, Figure 2), leading to different cationic/hydrophobic polymers with tunable antimicrobial activity [56,57]. These structural mimetics resemble the overall cationic amphiphilicity of HDPs rather than their typically defined secondary structures that create defined cationic and hydrophobic domains. 

To date, several types of synthetic antimicrobial polymers have been reported, and these may include but are not limited to poly-methacrylate copolymers, poly-norbornenes, nylon-3 copolymers, poly-carbodiimides, quaternary vinyl pyridines, and others. Some excellent reviews of the history, classifications, mechanisms of action, etc., of these cationic polymers were previously published [52,58,59,60,61].

### 2.2. Structural Features Influencing Bioactivity and Toxicity

#### 2.2.1. Cationic Functional Groups

Cationic groups are the sources of positive charge in polymers and are the components involved in the initial adherence of the polymer to the surface of the bacterial membrane through electrostatic interactions. A broad diversity of cationic groups in the monomers have been used in antimicrobial polymers, including primary, tertiary, or quaternary amine groups [62,63,64]; sulfonium ions [65]; phosphonium ions [66], etc. Inspired by lysine-rich HDPs, a primary amine group was one of the most commonly used cationic groups in polymers. In fact, several studies have revealed polymers bearing primary, secondary, or tertiary amines have relatively higher antimicrobial activity and lower hemolysis (lower red blood cell lysis) when compared to synthetic polymers bearing quaternary ammonium groups [63]. Polymers bearing primary amines have also been shown to have better activity than their tertiary and quaternary ammonium counterparts regarding bacterial cell surface binding and membrane-disrupting abilities [64,67].

Other forms of the cationic group in antimicrobial polymers include the guanidinium [68,69] and the iminium structures, such as pyridinium [70,71,72] and imidazolium salts [73,74]. It has been reported that iminium-containing cationic polymers exhibit relatively high antimicrobial activity (lower minimal inhibitory concentrations, MICs) against various bacteria and fungi when compared to counterparts with quaternary ammonium groups [75]. To date, many cationic monomers have been reported [56,76,77,78,79,80], and these studies concluded that cationic groups and their optimization in the polymer structure are required to achieve maximum antimicrobial activity with minimal toxicity against mammalian cells. 

#### 2.2.2. Hydrophobic Functional Groups

A complement to the cationic component, the hydrophobic monomers are the source of hydrophobicity in HDP-mimicking polymers. They are responsible for the polymer’s insertion into the lipid bilayer of the microbial membrane and disruption of membrane permeability (and presumably translocation across the membrane) [57,60]. Several hydrophobic group structures in the monomers have been used in antimicrobial polymers, including linear short alkyl groups (methyl, ethyl) or cyclic groups (cyclic hexane), etc. In general, similar to the cationic monomers, optimising the hydrophobic components is required to achieve maximum antimicrobial activity with limited toxicity towards mammalian cells. So far, linear alkyl groups have been the most effective hydrophobic group used in antimicrobial polymer, and their lengths significantly affect the polymers’ antibacterial activity [81]. For example, an earlier study by Hedrick et al. [82] investigated the influence of different sized lengths of alkyl chains in polycarbonate-based polymers, showing that MICs against pathogenic bacteria decrease as the alkyl chain length increases from 3 to 8 carbons [82]. Similar results were observed in other studies on different classes of antimicrobial polymers [83,84]. However, excess hydrophobic components or an increase in the length of the hydrophobic side chain can enhance the polymer’s hemolytic activity and decrease its solubility, which can be a challenging limitation. Consequently, in addition to the linear alkyl groups, fused and cyclic alkyl groups have also been investigated as hydrophobic monomers in the polymer mimics of HDPs. In fact, in some examples, the bacteriocidal activities of polymers bearing cyclic hydrophobic subunits were enhanced when compared to polymers bearing acyclic hydrophobic subunits [85,86,87]. 

#### 2.2.3. Hydrophobic/Hydrophilic Balance and Beyond

**Figure 3:**
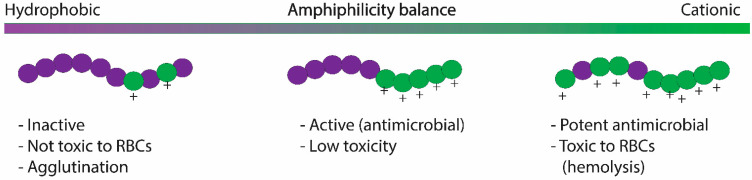
The diagram shows the influence of cationic/hydrophobic balance in the biological activity and toxicity of HDP-mimicking polymers.

There is substantial evidence that there should be some balance between the hydrophobic and the cationic monomers in the polymer structure to optimize the antimicrobial activity and decrease toxicity [52]. For example, excessive hydrophobicity increases hemolysis, i.e., toxicity, and produces poorly soluble polymers while generating highly potent antimicrobial polymers. In contrast, with excessive levels of cationic monomers, synthetic polymers are less but weaker antimicrobials in addition to increasing the tendency of polycations to lead to hemagglutination (aggregation of red blood cells (RBCs)). Consequently, the amphiphilic balance during polymeric design is critical to minimize the above limitations (see Figure 3 for illustration). Excellent discussion on the amphiphilic balance in HDP-mimicking polymers was reported in work by Ragogna P et al. [88], where they discuss the heterogeneous sources of amphiphilic balance in antimicrobial polymers. 

**Figure 4:**
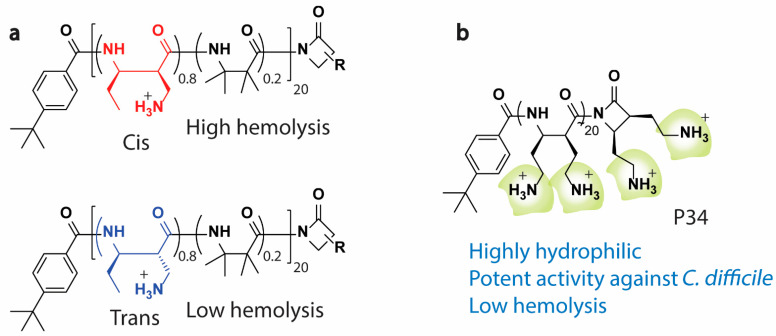
Beyond the amphiphilic balance of antimicrobial polymers. (**a**) Stereochemistry alters the activity of nylon-3 copolymer [89] and (**b**) an example of homopolymers with only cationic subunits (P34) but potent antibacterial activity and low toxicity against RBCs [90].

Beyond amphiphilic balance, several studies have revealed exciting results that could help re-formulate the way antimicrobial polymers are designed to improve activity. For example, a study by Gellman et al. found that changes in the polymer subunit stereochemistry alter the activity of nylon-3 copolymers [89]; in particular, stereoisomeric monomers bearing either a cis-aminomethyl side chain or a trans-aminomethyl side chain were created and tested for biological activity. The two stereochemical derivatives had an insignificant influence on the antimicrobial activity but demonstrated significant differences in hemolysis (Figure 4a) [89]. McBride et al. [90] also described a series of cationic homopolymers comprising only cationic subunits with strong efficacy against vegetative and spore forms of *C. difficile*. Interestingly, despite lacking the hydrophobic substituents of amphiphilic polymers, the compounds, especially P34 (Figure 4b), showed excellent efficacy against *C. difficile*, with low hemolytic activity and toxicity against intestinal epithelial cells [90]. Aside from this amphiphilic balance phenomenon, the effect of the polymeric block order on antimicrobial activity was also investigated [91,92,93,94]. A study by Boyer C et al. [91] concluded that by varying the combinations and order of polymeric blocks, the activity of HDP-mimicking polymers could be made tunable analogous to what has been shown for antimicrobial HDPs [91]. Thus, understanding the composition and particular arrangement of polymer synthetic blocks is essential for developing promising antimicrobial candidates with potent activities. Further discussions on the amphiphilic balance in HDP-mimicking polymers have been published [52,88,95]. 

#### 2.2.4. Introduction of Hydrophilic Functional Groups

**Figure 5:**
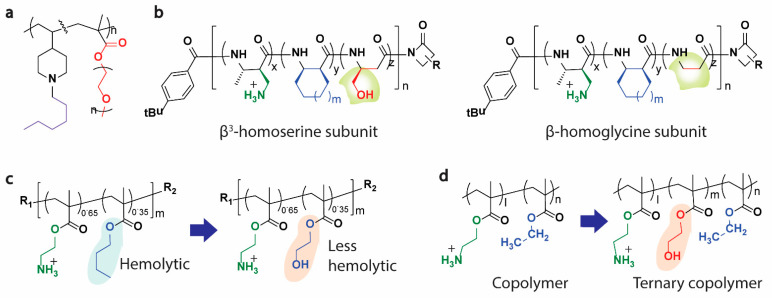
Ternary HDP-mimicking polymers. The introduction of neutral/hydrophilic groups/spacer, as shown, led to a significant reduction in hemolysis. (**a**) Poly-norbornene polymer incorporated with PEG [97], (**b**) introducing polar or uncharged units to the nylon-3 copolymers [98], (**c**) replacement of hydrophobic group with a hydrophilic group in a methacrylate-based polymer [99] and (**d**) introduction of a polar group as a spacer between the cationic and hydrophobic subunit of a methacrylate-based antimicrobial polymer [102].

Introducing hydrophilic subunits into the polymer to create an atypical structure has been shown to impact the amphiphilicity and the overall biological activity of antimicrobial polymers [96]. For example, when the molecular structure of poly-norbornene copolymer was incorporated with either zwitterionic, sugar, or polyethene glycol (PEG) moieties at various ratios, minor increases in the MICs were observed, whilst the hemolysis decreased significantly [97] (e.g., Figure 5a). Similarly, introducing a small proportion of polar or uncharged units to the nylon-3 copolymers alongside the cationic and hydrophobic subunits reduced hemolysis with minimal impact on antibacterial activity [98] (Figure 5b). In another study, a complete exchange of the hydrophobic groups with hydrophilic groups in a methacrylate-based polymer led to the maintenance of excellent antibacterial activity but significant reduction of their lytic effect against RBCs when compared to the original hydrophobic groups containing polymer [99] (Figure 5c). When introducing hydrogen bond donors or acceptors, the activity can change dramatically, as evidenced by increases in the activity of an N-alkyl maleimide-based amphiphilic antimicrobial polymer bearing an amide bond in the side chain when compared to a counterpart bearing an ester bond [100]. A similar study also demonstrated that the polymer bearing amide moieties requires fewer hydrophobic groups to create potent antimicrobials than the equivalent polymer containing subunits with an ester group [101].

Furthermore, a recent study by Kuroda et al. introduced a polar subunit (hydroxyl group) as a spacer between the cationic and hydrophobic subunit of a methacrylate-based antimicrobial polymer (Figure 5d), which decreased the hemolytic activity of the polymers significantly when compared to counterpart polymers with hydrophobic side chains [102]. Indeed, several studies have confirmed similar results in various polymeric backbones, suggesting that a ternary approach in polymeric design might be a promising strategy to optimize the activity and toxicity of the polymer mimics of HDPs [103,104,105,106]. 

#### 2.2.5. Molecular Weight

The polymer molecular weight is also considered an important parameter in designing highly active polymer mimetics of HDPs. Many studies have investigated the influence of the molecular weight on the activity of polymers; the majority concluded that the DP (degree of polymerization, which is the number of monomeric units in the polymer) or the molecular weight should be low for optimal bioactivity [53,54,107,108,109,110]. In some examples, however, polymers with longer chains have maintained or increased their antibacterial activities but concomitantly lead to increased hemolysis [53,111]. Hence, an appropriate molecular weight based on the type of the polymer is needed to achieve maximum activity with optimal biocompatibility.

Other features that influence the polymers’ overall biological activity may include the cationic charge density of the polymer, structural topology (distribution of components), terminal substituents, stereochemistry, and others [95]. Overall, extensive studies on the structure–activity relationships and factors influencing antimicrobial activity and toxicity of the polymers have been conducted and have contributed significantly to advancing the field of cationic antimicrobial polymers [56,112]. 

### 2.3. New Polymer Mimics of HDPs—Highlights

**Figure 6:**
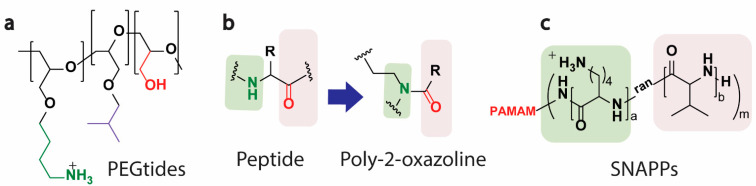
Examples of newly reported polymer mimics of HDPs. (**a**) mimicking amino acid residues on the backbone of the PEG chain and developing PEGtides, (**b**) glycine-like backbone substituent poly-2-oxazoline, (**c**) a new class of antimicrobial nanomaterials referred to as structurally nanoengineered antimicrobial peptide polymers (SNAPPs).

Searching for better polymer mimics of HDPs, Kim et al. [113] examined PEG and peptides, i.e., PEG-based peptides, as a novel mimic of HDPs (Figure 6a). Their study mimicked “key” amino acid residues found in HDPs, such as lysine, leucine, and serine, on a backbone of PEG chains and developed a series of PEG-based molecules that they termed PEGtides. Interestingly, several of these PEGtides possessed excellent activity against gram-negative and gram-positive bacteria while showing low hemolysis of human RBCs [113]. In another study using poly(2-oxazoline), Liu et al. [114] developed a glycine-like backbone substituent poly-2-oxazoline (Figure 6b) as a new synthetic mimic of HDPs. The compound exhibited potent in-vitro and in-vivo activity against methicillin-resistant *Staphylococcus aureus* (MRSA) and showed excellent activity in killing persister cells [114]. In another study, Qiao G et al. [115] described a new class of antimicrobial nanomaterials referred to as structurally nanoengineered antimicrobial peptide polymers (SNAPPs), which are star-shaped polymer nanoparticles consisting of lysine and valine residues (Figure 6c). Unlike conventional polymeric nanoparticles, the SNAPPs were considered stable unimolecular architectures and demonstrated sub-μM antibacterial activity against a wide range of clinically isolated gram-negative strains, including the ESKAPE (*Enterococcus faecium*, *Staphylococcus aureus*, *Klebsiella pneumonia*, *Acinetobacter baumannii*, *Pseudomonas aeruginosa*, and Enterobacter species) and colistin-resistant pathogens. In addition to high therapeutic indices, the SNAPPs displayed low toxicity towards mammalian cells [115].

A recent study by Silei Bai et al. [116] has also shown a short, amidine-rich antimicrobial polymer with dual-selective mechanisms of action against harmful “Superbugs”, including disrupting bacterial membranes and binding to bacterial DNA. This so-called oligoamidine showed high therapeutic indices against many bacterial types, including the ESKAPE pathogens and clinical isolates resistant to multiple drugs, with no observable resistance generation. Overall, such new strategies for designing polymer mimics of HDPs could be beneficial and could, indeed, open a new path towards developing innovative antimicrobial therapies.

### 2.4. Antibiofilm

Biofilms are formed when bacterial colonies (from single or multiple species) adhere to an abiotic or biological surface and embed in self-produced complex structures of extracellular matrixes [117,118]. Generally, biofilms are considered the typical form of bacterial growth in nature [117,118], are involved in 65% of all human infections, and are an alarming concern in the environment and industrial settings [119]. Biofilm infections are exceptionally challenging to treat relative to their planktonic (freely-swimming) counterparts since they are more highly resistant (by 10- to 1000-fold) to essentially all antibiotics and biocides and are refractory to host immune responses [119]. Unfortunately, to date, no drug has been approved that specifically treats biofilms, and usually, combinations of antibiotics are used. Several strategies, however, are under development, including the use of HDPs and their mimics, nucleotides, aptamers, bacteriophages, enzymes, engineered metal ions, and others [120,121,122]. 

**Figure 7:**
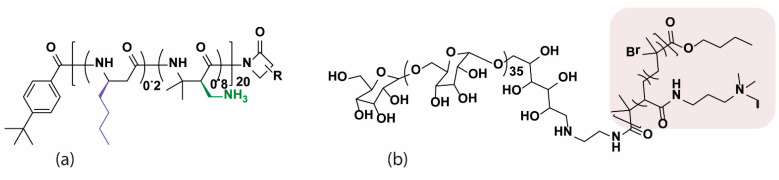
Examples of antibiofilm cationic HDP-mimicking polymers. (**a**) Cationic antimicrobial β-peptide polymer (20:80 Bu:DM) [123,124], and (**b**) a dextran-block methacrylate-based nanoparticle copolymer [128].

To contribute to the current fight against biofilm infections, researchers on cationic polymers have also investigated the feasibility of using these macromolecules to eradicate biofilms. For instance, we have recently shown that an amphiphilic poly-β-peptide polymer (20:80 Bu:DM, Figure 7a) has potent activity against biofilms of both gram-positive and gram-negative bacteria in addition to an interesting ability to modulate the innate immune response by stimulating chemokine and monocyte chemoattractant protein-1 (MCP-1) and suppressing the endotoxin-stimulated release of interleukin 1 Beta (IL-1β) from peripheral blood mononuclear cells [123]. In earlier studies, the polymer also showed potent antibacterial activity in vitro and in vivo [124] and was proven to effectively kill clinical isolates of gram-positive and gram-negative bacteria at concentrations that had very low toxicity towards mammalian cells [125]. 

Kuroda K et al. [126] reported the ability of methacrylate-based antimicrobial polymers to inhibit bacterial biofilms. The designed polymers showed potent activity against planktonic bacteria and inhibited biofilm development by cariogenic *Streptococcus mutans* [126]. The study was consistent with the study by Li et al. who showed inhibition of biofilms of *S. mutans* using cationic monomer methacrylate-based amphiphilic polymers. In a recent report, a pseudo-zwitterionic copolymer synthesized from mixed-charge subunits was also reported with excellent antibiofilm efficacy against MRSA and *Pseudomonas aeruginosa* [127]. Li J et al. [128] reported using dextran-block methacrylate-based nanoparticle copolymers with antibiofilm activity against MRSA, vancomycin-resistant *Enterococci* (VRE V583), and *Enterococcus faecalis*. Interestingly, their block copolymer (Figure 7b) diffuses into biofilms and attaches to bacteria but does not kill them; instead, it stimulates the gradual dispersal of biofilm bacteria [128]. An α/β chimeric polypeptide molecular brush (α/β CPMB) was also reported with excellent activity against biofilms of MRSA (inhibition and eradication). The polymer had further shown excellent killing of metabolically inactive persister cells, which are usually antibiotic insensitive [129]. There are quite a few other examples of antimicrobial polymeric materials targeting bacterial biofilms [130,131,132]; however, to our knowledge, the mechanisms by which these polymers exert their antibiofilm activities are not yet clearly understood. 

### 2.5. Biodegradable Cationic Polymers

**Figure 8:**
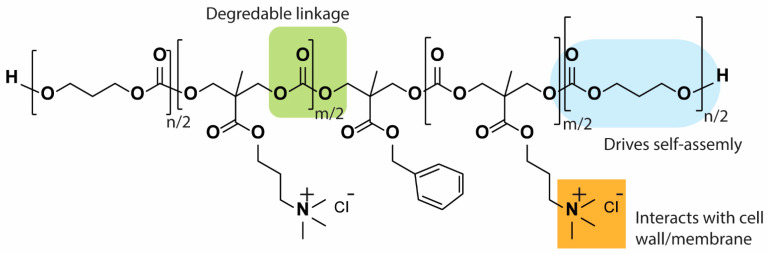
An example of biodegradable cationic amphiphilic polycarbonates-based polymer [133].

The majority of designed HDP-mimicking polymers tend to have non-degradable backbones, which may make them undesirable products in advanced preclinical and clinical studies. Undegradable molecules tend to accumulate in the body and cause long-term toxicity. In an effort to solve this drawback, some researchers have focused on designing biodegradable cationic antimicrobial polymers. For example, Yang Y et al. reported a biodegradable antimicrobial polymeric nanostructure based on functional cyclic polycarbonates exhibiting excellent selective antimicrobial activity towards the bacterium MRSA and fungi (Figure 8) [133]. 

In another study, Yang Y et al. [134] reported biodegradable antimicrobial polymers of guanidinium-functionalized polycarbonates with potent bactericidal activity and a distinct mechanism that does not allow the induction of drug resistance. In addition, the designed polymer was nontoxic and exhibited broad-spectrum, in-vivo antimicrobial activity. Several other examples of biodegradable cationic polymers have also been reported, and all have shown excellent activity with good biocompatibility [135,136,137,138]. Indeed, these degradable cationic antimicrobial polymers, which lead to nontoxic degradation by-products in vivo, offer promising, safe polymeric antibacterial macromolecules.

### 2.6. Advantages: HDPs and HDP-Mimicking Polymers 

HDPs have attracted considerable attention in the last decades as potentially new antimicrobial drugs due to their benefits. (1) Peptides exhibit a low propensity to develop bacterial resistance, and their physicochemical properties and chemical structures are considered evolutionarily optimized to selectively target bacteria [139]. The mechanism by which HDPs act can involve multiple targets, including bacterial cell membranes, leading to complete damage of the membranes and cell death in addition to cell wall synthesis, cell division, and/or a range of intracellular targets, including compromising macromolecular synthesis (DNA/RNA/proteins) [21]. It is very challenging for the bacteria to adapt a resistance strategy towards these multifaceted mechanisms, although some resistance mechanisms to HDPs influencing, for example, uptake or stability have been reported [23,139]. (2) Peptides are generally biodegradable molecules and do not persist in the body or the environment. Biodegradability is both a compromising issue (if the molecule is removed too rapidly) and essential for drug design since it can decrease toxicities by helping to prevent long-term accumulation of medications in the body. In addition, it is important to the environment to reduce exposure of the peptides to environmental microorganisms for an extended period, especially at low concentrations that can lead to an emerging pool of resistant bacteria [139]. (3) Unlike antibiotics, where the antimicrobial activity can be relatively selective, peptides often have a broad spectrum of activity, targeting a large number of strains from both gram-positive and gram-negative pathogens. This makes them excellent candidates for clinically accepted therapies. (4) The ability of some HDPs to act against recalcitrant bacterial biofilms is perhaps one of the most significant advantages of such HDPs over conventional antibiotics, which work poorly against biofilms even when applied in combinations. (5) The ability of HDPs to modulate the immune system, a recently appreciated functionality of HDPs, make these small molecules attractive candidates as anti-infective agents with an ability to selectively stimulate protective immunity while suppressing inflammation [13]. 

HDP-mimicking polymers have elicited substantial interest in combating drug-resistant bacteria since they possess some or all of these advantages of HDPs in addition to having certain additional benefits of biocidal disinfectants. Thus, (1) like HDPs, antimicrobial polymers can have broad-spectrum activity against gram-positive and gram-negative bacteria, including the hard-to-treat ESCAPE pathogens. In addition, some have demonstrated excellent activities against other microorganisms, including fungi. (2) Low susceptibility to develop bacterial resistance was observed with many polymer mimics of HDPs. (3) Polymers are generally stable against metabolic enzymes and provide stable macromolecules in long-term usage and storage. (4) Unlike peptides, where large-scale production can be difficult and expensive, polymer chemistry can provide easy and cost-efficient methods for large-scale polymer production [140,141,142].

### 2.7. Limitations: HDPs and HDP-Mimicking Polymers 

HDPs are excellent small molecules to be developed as drug therapies to reinforce the anti-infective arsenal. Nevertheless, and despite the abovementioned benefits, the transition of these molecules into clinical use is impeded by a number of constraints. (1) Peptide production by either chemical synthesis, peptide expression/isolation from recombinant microbes, or biological display technologies are costly and time consuming, and the latter two technologies are not developed for large quantities. (2) Peptides generally have poor stability to proteases and peptidases, leading to loss of activity. (3) Some HDPs are quite toxic to mammalian cells, especially in vivo; one type of toxicity is driven by the tendency of amphipathic cations, like peptides, to aggregate [143]. Further studies, in fact, are needed to address this issue and understand in depth the mechanism(s) by which toxicity occurs. 

By analogy, while cationic polymers were designed to mimic the physicochemical features of HD and overcome some of their limitations, they can also present certain weaknesses that need to be addressed before transitioning them into clinical antibiotics. For example, (1) as observed for HDPs, polymers often possess significant toxicity to eukaryotic cells, which may be related to their physicochemical properties and somewhat non-selective mechanism of action. While most investigations have attempted to address this issue and have succeeded to some extent in many cases, most were at the cost of losing or reducing antimicrobial activity. Therefore, there is a persistent need to develop antimicrobial polymeric compounds with broad-spectrum activities against microbial cells that exhibit limited or no toxicity towards eukaryotic cells. (2) Polymers often have high molecular weights; thus, they may demonstrate poor aqueous solubility, leading to low antimicrobial activity. The molecular weight can indeed affect both solubility and the activities of the polymers as it influences the size, net charge, and hydrophobicity [53,144,145]. In addition, large macromolecules may not act as rapidly as small molecules, such as oligomers or peptides, against microorganisms. (3) Several cationic polymers designed to mimic HDPs are to date neither biocompatible nor biodegradable, and their use would likely be restricted to use on surfaces through integration with appropriate materials either by self-assembly or grafting to produce formulations with adequate performance and low systemic or in-vivo toxicity. Nonetheless, it is worth stating that the progress in the area has led to the discovery of some polymers with better biocompatibilities [86,114,134,138,146], and alternative approaches that could solve this issue, such as the design of biodegradable polymers and large polymeric macromolecules [115,147,148]. (4) While numerous antimicrobial HDPs have been reported in the literature, only small numbers have proceeded into clinical trials [33,149], and none have made it to the marketplace (although cationic amphipathic peptides, like gramicidin S, polymyxin B, and nisin, have been successfully commercialized in the past). Similarly, to date, no polymer mimics of HDP have reached this benchmark, and to our knowledge, extensive studies are needed before these polymers can be introduced to clinical use.

### 2.8. Potential Success of HDP-Mimicking Polymers

Microbial infections, including bacteria, threaten human health and economic stability, and without doubt, new medications and alternatives to failed/failing antibiotics are needed. HDPs have a significant promise to supplement antibiotics in tackling infectious diseases and reinforcing the anti-infective arsenal. Likewise, HDP-mimicking antimicrobial polymers have an enormous potential to supplement antibiotics. The potential success of these macromolecules arises from their advantageous properties, including their broad-spectrum activities, low susceptibility to bacterial resistance, chemical stability, and the ease of large-scale production at modest costs. Nevertheless, further studies are needed before initiating clinical development leading to approved therapeutics. 

First, while a wide diversity of chemical structural studies has been explored, precise knowledge of polymer chemistry and the structural features that regulate anti-infective activity is still lacking. A complete understanding of the underlying mechanisms responsible for bioactivity and toxicity is crucial for designing polymeric compounds that can recognize prokaryotes from eukaryotes. Hence, we favour the idea of revisiting the fundamental design principle of cationic polymers, and we see it as a challenging but promising approach towards taking full advantage of HDPs and their analogs. The current structural design of cationic polymers relies on incorporating two main components, cationic and hydrophobic subunits, at different ratios and then optimizing the length, charge density, amphiphilicity, etc., to achieve a preferable model with minimal toxicity and maximal activity. Contrarily, HDPs encompass various amino acids (in addition to the cationic and hydrophobic residues) and cover a significant to a vast area of chemical space that significantly influences each peptide’s activities [23]. This suggests that cationic antimicrobial polymers, due to their innate simplicity, have not yet taken full advantage of the physiochemical properties available for HDPs. 

Second, many HDPs have recently been shown to synergize with conventional antibiotics; this provides another path for their practical use in addressing bacterial resistance mechanisms [150,151,152]. For example, peptides can increase the intracellular concentration of antibiotics by increasing their cellular uptake based on several studies performed against planktonic bacteria [153,154,155]. Furthermore, this synergistic or additive effect has also been demonstrated to enhance the antibiofilm activity of HDP/antibiotic combinations [156,157,158]. Indeed, the synergy enables the targeting biofilms of difficult to treat pathogens (e.g., ESKAPE pathogens) in in-vivo models [159,160]. Similarly, in addition to monotherapy, cationic antimicrobial polymers might be used alternatively as adjuvant therapies in combination with antibiotics. While there are limited data on this approach, a few studies have reported exciting results. Thus, Zangeneh R et al. showed a synergistic/additive effect of a combination of a synthetic HDP-mimicking polymer (oligoethylene glycol, hydrophobic ethyl hexyl, and cationic primary amine groups) and two antibiotics, doxycycline and colistin, against *Pseudomonas aeruginosa* and *E. coli* [161]. In another study, a vitamin E-functionalized cationic polycarbonates polymer co-delivery with doxycycline showed an excellent synergistic effect towards *Pseudomonas aeruginosa* [162]. Synergistic activity between a cationic antimicrobial polycation (polyacrylamide) and daptomycin was also reported against *Staphylococcus aureus* biofilm [163]. Several combination strategies involving synthetic antimicrobial polymers were discussed previously [164], emphasizing combinations with nitric oxide, antibiotics, essential oils, and metals. These synergistic data reportedly showed the potential of coadministration of antimicrobial polymers with antibiotics. Thus, such synergistic interactions will improve treatment effectiveness and provide a practical approach to extending the lifetime of antibiotics by restoring the susceptibility of the multidrug-resistant bacteria to antibiotics. 

Third, while extensive studies have been performed on amphiphilic antimicrobial polymers to optimize their activity, limit toxicity, and address structure-activity relationships [54], very minimal but encouraging studies have tested the efficacy of the cationic polymers in animal models. Some of these studies include, for example, testing cationic methacrylate-based polymers in vivo against *Staphylococcus aureus* nasal colonization, where the polymer demonstrated a significant reduction in the number of viable *Staphylococcus aureus* in the nasal environment of cotton rats relative to controls [108]. Other topical uses of the polymers demonstrated activity in burned skin [101], wound healing [165], abscess infections [123], and the cornea [166]. When given by injection, cationic polymers in a few other studies also demonstrated good compatibility and high efficacy in treating multiple multidrug-resistant infections [115,134,167,168]. These referenced in-vivo experiments and perhaps others are certainly adding significant additional information regarding the potential therapeutic applications of HDP-mimicking polymers. 

Last but not least, toxicity, pharmacokinetics, and dynamic studies must be carried out for HDPs, and their polymer mimics in ex-vivo, human tissue models as well as in animal models to determine their systemic acceptability and compatibility and identify potentially serious side effects, to guide future drug design and development. 

## 3. Polymer Brushes 

Infection of medical devices and biomaterials is a major healthcare burden, which often leads to increased hospitalization, duration of stay at the intensive care units, cost of treatment, and high morbidity and mortality rates [169]. In recent years, polymer brushes-nanofilms of polymeric materials grafted onto inert surfaces have attracted considerable attention as a novel strategy to fight infections, including preventing the formation of biofilms associated with various medical devices, such as catheters, heart stents, dental implants, orthopaedic implants, contact lenses, etc. Grafting or, in other words, “coating” these devices with polymer brushes can provide antifouling activity by preventing bacterial adherence onto the surfaces and mediating antimicrobial activity. They provide ideal surfaces that can improve the long-term performance and stability of devices/implants. Various polymeric brushes have been designed on multiple substrates during the last decades [170,171,172,173], and here, we feature some examples of the supramolecular assemblies of polymer brushes with HDPs.

### 3.1. General Approaches for Surface Coating 

Surface coatings can be classified into three different types based on the strategy used to protect against bacterial infections, including (1) antifouling-based coatings representing an antiadhesive approach. In this category, hydrophilic biomaterials (or polymers) are anchored onto the surfaces to discourage or reduce the capacity of bacterial adhesion onto the substrates. While the approach prevents surface contamination, it does not kill bacteria or inhibit their growth in solutions. Examples of such biomaterials are PEG and polymers with a zwitterionic nature, such as poly-(phosphorylcholine), poly(sulfobetaine), and poly-(carboxybetaine) [174,175]. (2) The second method is biocidal release-based coatings, in which the surface-coated biomaterials release biocidal compounds over time into the surrounding environment, killing adhered and nearby bacteria adjacent to the device. Various biocidal and antimicrobial agents have been used, such as antibiotics, nanoparticles, metals, bacteriophages, etc. [176,177,178]. (3) The third method is contact killing-based coatings. In this strategy, an antimicrobial agent is attached to the surfaces that act as an antimicrobial and hopefully an antibiofilm agent. Using this approach, the bacteria are often killed upon contact although this strategy’s limitation is that the device can become coated with dead bacteria, providing a surface that does not mediate contact killing. Various biomaterials have been anchored on multiple substrates, including conventional antibiotics, HDPs, HDP mimetics, and others [179,180]. 

### 3.2. Surface Engineered Polymeric-Brush-Tethered HDPs

Anti-adhesive agents in surface modifications (i.e., hydrophilic polymer brushes) provide coated surfaces that prevent initial bacterial adhesion; however, such architectures do not kill or inhibit biofilm growth, and the few adhered bacteria can over time still form a mature biofilm [181,182]. Therefore, to overcome this shortcoming and improve surface coatings, surface-anchored polymer brushes have been functionalized with antimicrobial agents to kill bacteria and inhibit biofilm development [32,182,183,184,185]. 

**Figure 9:**
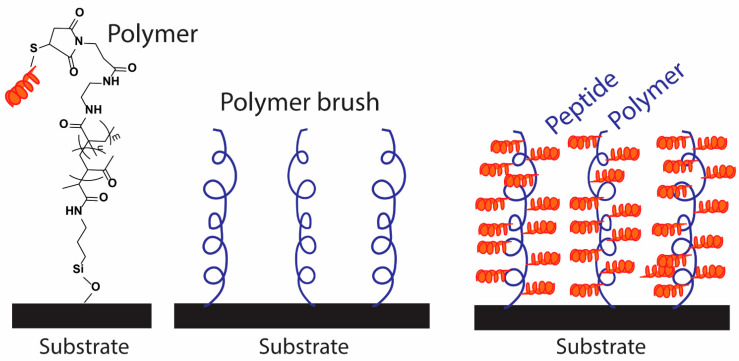
Representation of polymer brush integrated HDP on a substrate.

Among these antimicrobial agents, HDPs exhibit appealing features for designing antibiofilm surfaces (Figure 9). Indeed, integrating HDPs onto polymer brushes can provide several substantial benefits to the coating materials (or polymers), such as (1) creating highly hydrated (amphiphilic) surfaces with antifouling and antimicrobial properties, (2) providing broad-spectrum activity against a wide range of bacterial species, (3) inhibiting biofilm development, and (4) generating highly biocompatible materials in vivo. In addition to these positives, HDPs are also considered viable alternatives to antibiotics due to their low susceptibility to develop resistance, and relative to biocides, HDPs tend to have lower toxicity towards mammalian cells with negligible immunogenicity.

The implications of integrating HDPs to polymer brushes have certainly enhanced the coated surfaces’ antimicrobial and antibiofilm performance. Numerous reported studies by our research group and others suggested that the conjugation strategy of HDPs onto polymer brushes is highly effective in preventing bacterial adhesion and biofilm development on various surfaces in vitro [183,186,187,188,189,190,191]. Indeed, the approach has also been proven to be efficient in in-vivo models. For example, in a recent study, polymer brushes integrated with HDPs on polyurethane, a common material used for catheter manufacture, prevented bacterial adhesion by 99.9% (for both gram-positive and gram-negative strains) and inhibited planktonic growth by 70% in vitro. When tested in a mouse urinary catheter infection model, the HDP-conjugated polymer brush catheters demonstrated reduced the bacteria adhesion onto the catheter surface by more than 4 logs when compared to the uncoated catheter surfaces [192]. In another study by Fang Z et al., polymer brushes with mono- and dual-peptide functionalized Ti rods implanted in a rabbit femur (bone) defect model exhibited excellent biocompatibility and antimicrobial activity [193]. Several other studies have reported in-vivo experiments [194,195,196,197,198] reinforcing the promise of using polymer-brush HDP-conjugate design to prepare functional biomaterial surfaces. In addition to their conjugation with standard polymer brushes, peptides with antimicrobial activity have also been integrated into or with other films/surfaces, such as protein-based films (i.e., self-assembled protein nanostructures) [199,200,201,202,203,204,205,206], silk surgical sutures [207,208], metal nanoparticles [209,210] and other solid interfaces, in order to provide biomaterials that can prevent and minimize the risks of bacterial infections. 

It is worth noting that aside from intriguing contributions to surface science, integrating polymer brushes into HDPs in solution represents a novel advanced approach that provides significant advantages to HDPs, such as enhancing therapeutic performance, decreasing toxicity towards mammalian cells, and protecting peptides from degradation by digestive enzymes, as discussed in several representative reviews [211,212,213,214,215] of the therapeutic potential of HDP–polymer conjugates.

### 3.3. Challenges and Future of Polymeric-Brush-Tethered HDPs

Despite the advantages offered by the polymeric-brushes HDP-conjugation strategy in coating surfaces of medical devices and implants, there are a number of challenges and constraints that need to be fully addressed. For example, (1) retaining sufficient activity of the immobilized peptides is not an easy task, and there is no formal relationship between the activity of HDPs when bound and free [216]. In fact, the activity of the bound peptides is influenced by many intra- and interlinked factors, such as the structure of the peptide itself, sequence length, type of surface coated, the density of the peptide on the surface [216], spacer length [217], composition, molecular weight, and many others. Moreover, (2) other factors, such as the nature of the surrounding environment pH and ionic strength (i.e., salt concentration), which vary based on body location, can significantly impact the performance of the coated layers on the device or implant. Investigating these influential factors case by case must be considered when studying the activity of bound HDPs; nevertheless, the process can be very tedious, time consuming, and unfeasible for high-throughput screening. (3) Theoretically, the stability of HDPs might be a limitation in the described strategy since peptide degradation or denaturation might lead to a loss in antimicrobial surface activity. Alternatively, for unstable peptides, synthetic analogs or HDP mimetics can be used to circumvent this limitation. (4) Accumulation of dead bacteria on the antimicrobial coated surface can also be a challenging problem that needs to be solved. Immobilized antimicrobials catch bacteria and kill them, but those dead bacterial cells plus debris remain on the surface and act as a second layer (a breeding ground) for other bacteria to grow, rendering the coated surface ineffective [218]. While tethered HDPs on polymer brushes minimize the issue to some extent, they do not prevent accumulation. One strategy to overcome this is to use layers with self-cleaning properties or combine antifouling and antimicrobial properties into the polymer brush strategy to repel bacteria and those killed by the surface-exposed HDPs [219,220]. However, we must be concerned whether the antifouling properties of the coated surfaces come at the expense of reducing antimicrobial activity. Further studies are needed in these directions.

Despite the limitations mentioned above, the available data on polymer brushes is encouraging, and we believe that integrating HDPs into the polymers is a significant step toward addressing a clinically important problem. Indeed, the strategy is one of the most promising approaches to avoid or minimize bacterial colonization on surfaces of medical devices and implants. 

## 4. General Conclusions

This review discusses polymeric biomaterials, including HDP-mimicking polymers and polymeric brushes tethered to HDPs, as two advanced strategies to tackle infectious diseases. Cationic antimicrobial polymers are certainly one of the best possible candidates for surmounting certain constraints of HDPs, including lack of stability and cost of synthesis. The ease of synthesis and access to a wide variety of synthetic materials provide substantial incentive to continue to develop such polymers. In addition, the increase in the development of degradable polymeric biomaterials makes it increasingly possible to create products that can avoid toxicity and persistence in the environment. Similarly, polymer brushes integrated with HDPs offer a consistent and highly effective approach to fighting infections associated with indwelling medical devices and implants. While systematic analysis and in-depth understanding of the peptide-coating structure: activity relationships are required to establish robust strategies to produce efficient antimicrobial surfaces, the success of this approach will make a significant contribution to addressing challenging issues associated with nosocomial infections and biofilms.

## Data Availability

No new data were created or analyzed in this study.

## Abbreviations

HDP	Host defense peptide
AMP	antimicrobial peptide
PEG	polyethene glycol
SNAPPs	structurally nanoengineered antimicrobial peptide polymers
*C. difficle*	*Clostridium difficile*
RBCs	red blood cells
MRSA	methicillin-resistant *Staphylococcus aureus*
VRE	vancomycin-resistant *Enterococci*
ESKAPE	*Enterococcus faecium*, *Staphylococcus aureus*, *Klebsiella pneumonia*, *Acinetobacter baumannii*, *Pseudomonas aeruginosa*, and *Enterobacter* species
MIC	minimal inhibitory concentration
MCP-1	Monocyte chemoattractant protein-1
IL-1β	Interleukin 1 Beta
